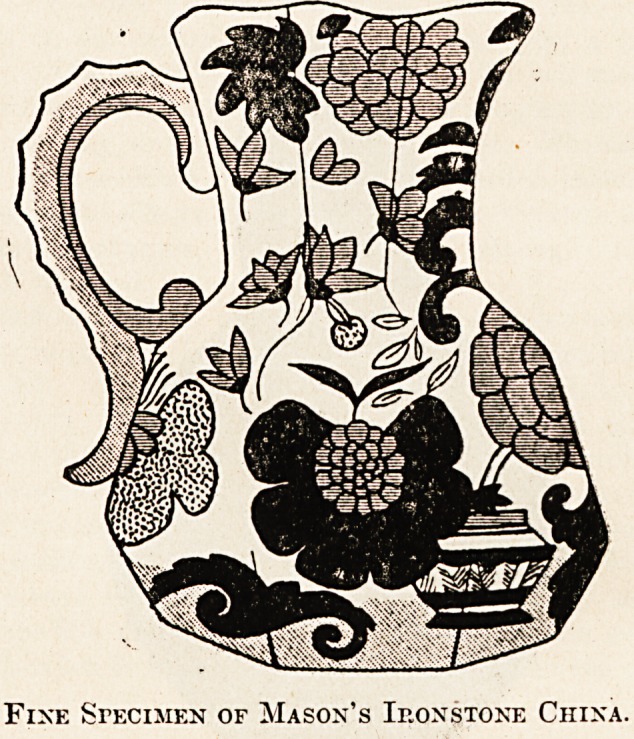# Old China

**Published:** 1907-08-03

**Authors:** W. Heneage Legge

**Affiliations:** Ringmer, Lewes


### The Practitioner's Relaxations and Hobbies.

### OLD CHINA.

By W. HENEAGE LEGGE, Ringmer, Lewes.

Although the hobby of collecting what is commonly called "old china" is more related to art than to science, it is not the less a recreation suitable to the doctor as a man of culture. Nor is it altogether destitute of scientific interest, as it includes within its purview matters having a definite connection with archæology, though these are not objects with which the collector usually concerns himself. So much the better for those of the wider view, who find the interest of the subject the more enhanced by its extension backwards into the realm of the past, where history has to be sought in the works of man's hands as evidences of his condition and attainments.

"Greybeard" Found in Ringmer.

### The Earliest Examples.

There, in the most remote periods of the prehistoric age, we find abundant witness to the antiquity of the potter's art—an antiquity so great that in all probability man's first efforts in the region of art were endeavours to mould the plastic clay into forms pleasing to his eye as well as useful to his needs, or to trace on its soft surface those simple patterns that still survive on sepulchral urns or domestic utensils. For most ancient sepultures brought to light are associated with fictile objects, many of which exhibit ornamental lines or attempts at more elaborate patterns. The collector, too, need fear no great difficulty in procuring specimens of prehistoric pottery. If not fortunate enough to discover such relics himself, he can often purchase them quite cheaply from dealers' shops.

### Roman Work.

As we advance in time to the days of the ancient Greeks and Romans we find the potter's art in such perfection that no higher forms of beauty and skill can be desired than the best of their work in clay, porcelain, or glass. In comparison with those times the Middle Ages suffer, though in some other domains of art they are far from being so uninspired or unskilful as they are usually depicted. Even in branches of the potter's art they produced works of originality and worth, as exemplified in great numbers of beautiful encaustic, inlaid, and glazed tiles used in ecclesiastical edifices, the best of which were made in this country.

### Renaissance Pottery.

With the dawn of the Renaissance ceramics blossomed with great vigour, and Palissy produced his beautiful fayence. In the Netherlands the potters turned out great quantities of their domestic wares, particularly those strong "stone" jugs called "Bellarmines," presenting a caricature likeness of the Cardinal of that name, then much in evidence as a theological controversialist. It is possible these jugs were also produced in England, for they are certainly quite common here. I possess two, found under the floors of some old cottages when they were demolished. The accompanying illustration well shows the face of the Cardinal with his long beard—whence the alternative name for these jugs of "greybeards"—and his very portly priestly form. Their usual height is about eight or nine inches, and they may be bought for something less than a sovereign; some are much larger, and I have seen one nearly two feet

Old Fulham Pot, Ringmer.

high, which sold for a five-pound note, albeit perfectly plain except for a small medallion of impressed ornament, no larger than a penny-piece.

### English Ware.

I have said it is possible such wares were made in England, for there were abundant potteries about this country, even at an earlier date. A group of potters' and brickmakers' kilns was unearthed in my parish some years ago, and fragments of their wares were found in abundance, mainly of a plain character, though the presence of such a portion of a jug as that depicted here shows that other more ornamental designs were attempted. In the court rolls of the manor I have found references to its potters as far back as Edward III.'s reign.

### Lambeth and Fulham Ware.

Another kind of old stoneware which is by no means rare is that of the Lambeth and Fulham potteries. The latter were established about 1630; the former somewhat earlier. The Fulham ware has a kind of family likeness to the Bellarmine pottery, and is usually met with in the form of jugs, very strong in substance, grey in colour, with bold blue, somewhat rude, patterns of scroll, leaf, or flower; often with a medallion of the royal initials impressed on the front of the jug; almost always with sunk lines encircling the neck, the meaning of which is unknown, but which appear to me to be intended to facilitate their being mounted with silver, or the attachment of a lid. The fine specimen here illustrated is of Georgian date, and once belonged to the mess of an Artillery regiment quartered in the aforesaid village.

After Fulham came a greatly agumented output of the Staffordshire potteries, andWedgwood raised

Portion of the Spout of a Jug Found Among Debris at the Mediæval Pottery at Ringmer.

the potter's art to great excellence by his originality and good taste. It is well to know that, in so far as the age of any piece of this ware adds to or determines its value, the mark (usually impressed) "Wedgwood and Bentley" fixes its date as prior to 1787, since this partner last named left the firm in that year. Some years ago I was given a beautiful little oval medallion of Wedgwood ware, representing the Farnese Hercules in high white relief on the well-known pale-blue ground, and marked, at the back, in this manner. It had been bought for sixpence in the East End, a chance purchase, since its nature was indistinguishable in black dirt.

### Staffordshire.

Nowadays "Staffordshire" of various firms and makers, is the particular ware of "old china"

Fine Specimen of Mason's Ironstone China.

most likely to come under the view of the collector, on account of the abundant output of the potteries of that county and neighbouring districts. Some of these are now extinct, and specimens of their ware becoming somewhat rare. *Cœteris paribus*, the age of any particular piece makes, to a great extent, its value, and it is interesting to compare the early specimens of a particular pattern or design with those of present manufacture. Blue or blue and red coloured patterns were favourites with these potteries, and reproductions or modifications of the "willow pattern" were abundantly made. "Mason's ironstone china" in red and blue designs is a pleasing form of old ware frequently seen in jugs of polygonal sides, as this illustration of a very pretty specimen will show. A similar shape was also used by Davenport. Of other china manufactories, specimens of old Derby and Chelsea may still be picked up, with the anchor or the crown mark to denote their origin; though, of course, other wares may bear very similar marks or modifications of them, as the anchor of Devonport, the crown of Worcester. A deep rich blue and much gilding was used in the latter ware, some of which is very beautiful and valuable. Early pieces often have marks like Chinese or Japanese characters; and, indeed, the decoration of many specimens is very Oriental in design, if not actually so in execution, since in olden days porcelain of English make was sent out to the East to be decorated in the style of the Orient, in accordance with the fashion then much in vogue. Such is a brief glimpse of a hobby which is as fascinating as it is varied in the objects of the collector's pursuit.

## Figures and Tables

**Figure f1:**
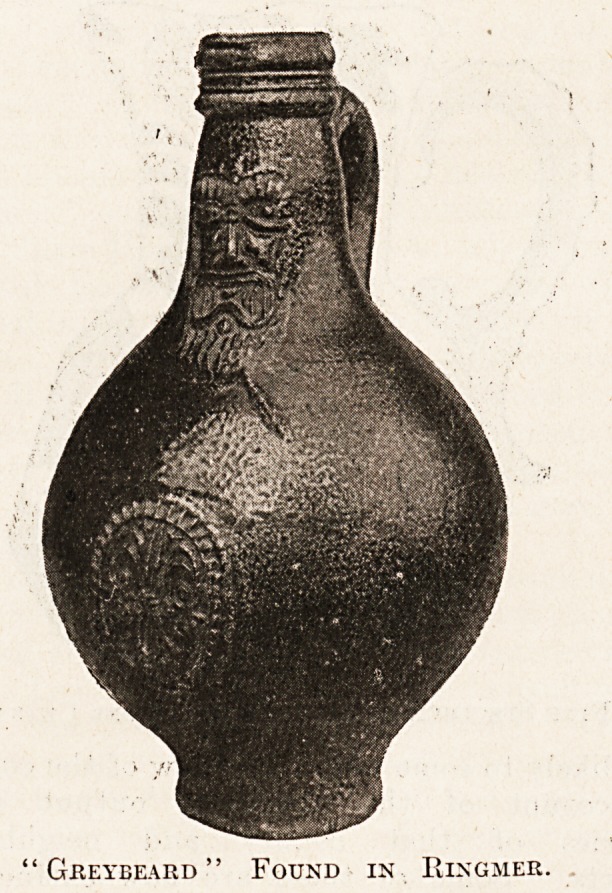


**Figure f2:**
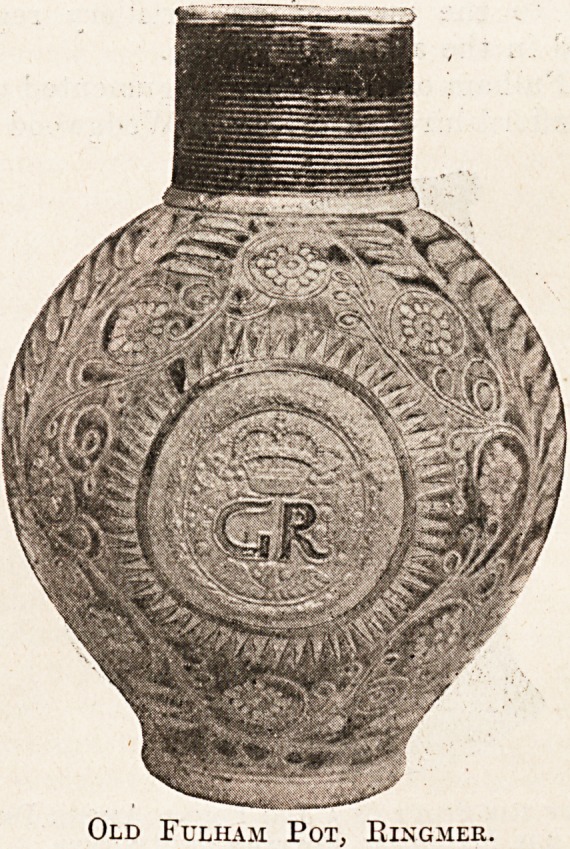


**Figure f3:**
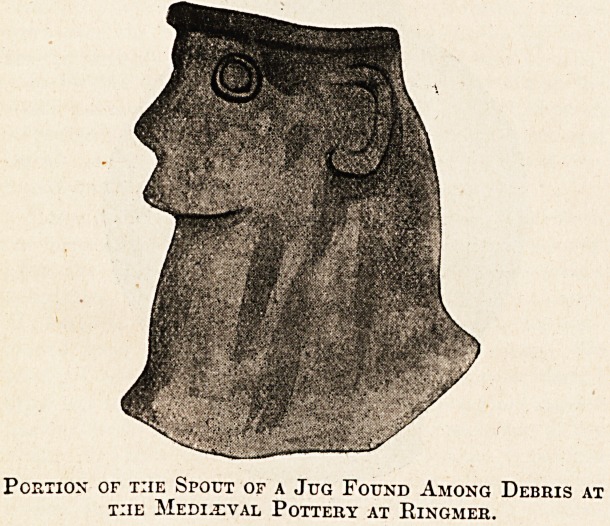


**Figure f4:**